# Macrophage Migration Inhibitory Factor Levels in Gingival Crevicular Fluid, Saliva, and Serum of Chronic Periodontitis Patients

**DOI:** 10.1155/2019/7850392

**Published:** 2019-02-05

**Authors:** Yveth Marlene Ortiz-García, Trinidad García-Iglesias, Gabriela Morales-Velazquez, Blanca Patricia Lazalde-Ramos, Guillermo Moisés Zúñiga-González, Ramón Guillermo Ortiz-García, Ana Lourdes Zamora-Perez

**Affiliations:** ^1^Doctorado en Ciencias Biomédicas, Centro Universitario de Ciencias de la Salud, Universidad de Guadalajara, Guadalajara, Jalisco, Mexico; ^2^Instituto de Investigación en Odontología, Centro Universitario de Ciencias de la Salud, Universidad de Guadalajara, Guadalajara, Jalisco, Mexico; ^3^Laboratorio de Inmunología, Centro Universitario de Ciencias de la Salud, Universidad de Guadalajara, Guadalajara, Jalisco, Mexico; ^4^Unidad Académica de Ciencias Químicas, Universidad Autónoma de Zacatecas, Zacatecas, Mexico; ^5^Laboratorio de Mutagénesis, Centro de Investigación Biomédica de Occidente, Instituto Mexicano del Seguro Social, Guadalajara, Jalisco, Mexico

## Abstract

Chronic periodontitis (CP) is an infection that affects the teeth supporting structure. Macrophage migration inhibitory factor (MIF) is an important effector cytokine of the innate immune system. Due to its functional characteristics, MIF may be involved in the immunopathology of CP. The aim of the present study was to evaluate MIF levels in gingival crevicular fluid (GCF), saliva, and serum of CP patients. A cross-sectional study was conducted on 60 subjects divided into two groups: subjects with CP (n= 30) and periodontally healthy subjects without CP (n=30). MIF was quantified in GCF, saliva, and serum of all participants by enzyme-linked immunosorbent assay. MIF concentrations were higher in GCF, saliva, and serum in the group with CP compared with the group without CP and a higher MIF concentration was observed in GCF (p=0.001) and saliva (p=0.009) in the group with CP. MIF intragroup comparisons between fluids demonstrated significant high levels of MIF in saliva compared with GCF and serum in both study groups (p<0.05). A positive correlation was found between clinical signs and MIF concentration in GCF (p<0.05). There is an association between the MIF and the clinical signs of the disease. Therefore, MIF could have an important role in the pathology and progression of CP.

## 1. Introduction

Chronic periodontitis (CP) is an inflammatory disease characterized by the loss of periodontal tissues that support the dental organs [1]. The presence of biofilm and dental plaque initiates a destructive inflammatory process that leads to the formation of periodontal pockets [2, 3]. This represents a well-characterized microbial change during the transition from periodontal health to periodontal disease [4], primarily by the presence of key pathogens, such as* Porphyromonas gingivalis*,* Tannerella forsythia*, and* Treponema denticola *[1, 4, 5].

The bacterial components of periodontopathogens, such as lipopolysaccharides (LPS), peptidoglycans, lipoteichoic acid, proteases, and toxins, induce inflammation [6]. This inflammatory response activates cells and mediators that contribute to tissue degradation and bone resorption [7].

Cytokines are an important component of the inflammatory process in CP. It has been shown that proinflammatory cytokines such as IL-1, IL-6, IL-17, and TNF-alpha are involved in the immunopathology of this disease [7, 8].

Macrophage migration inhibitory factor (MIF) is an important effector cytokine of the innate immune system [9]. MIF is constitutively expressed by a variety of immune and nonimmune cells (e.g., eosinophils, neutrophils, monocytes/macrophages, lymphocytes, endocrine, endothelial, and epithelial cells) [10]. MIF promotes the migration and recruitment of leukocytes in the sites of infection and inflammation and it is rapidly released in response to stimuli like microbial products, proliferative signals, and hypoxia [11].

MIF regulates the innate immune response through modulation of the receptor recognition pattern, such as TLR4, and through increasing the expression of cytokines, such as TNF-alpha, IL-6, and prostaglandins E2 [12–14].

Studies have shown that MIF is an enhancer of osteoclastogenesis by upregulating the signaling pathway of NF-kB in a periodontitis model [15] and a rheumatoid arthritis model [16] in mice.

Previous research has shown that MIF can participate in the pathophysiology of chronic degenerative and autoimmune diseases by significantly increasing body fluids, as in rheumatoid arthritis [17], diabetes type 1 and 2 [18, 19], metabolic syndrome [20] and oral squamous cell carcinoma [21], among others. Due to its functional characteristics, MIF may be involved in CP immunopathology. Previous studies have indicated the presence of this cytokine in epithelial tissue of patients with CP [22], in gingival crevicular fluid (GCF) of subjects with induced gingivitis [23], and in individuals with aggressive periodontitis [24].

At the present time, the concentration of MIF in GCF, saliva, and serum from subjects with CP is unknown. Consequently, the aim of this work was to determine and compare the GCF, saliva, and serum concentrations of MIF in individuals with CP and healthy subjects and to correlate MIF concentrations with periodontal clinical parameters in individuals with CP.

## 2. Materials and Methods

### 2.1. Study Population

This is a cross-sectional study that was conducted in the Periodontal Clinic, Dentistry School of the University of Guadalajara.

The study was carried out in 60 participants (30 males/30 females; mean age 40.64 ± 9.67 years) and all participants provided informed written consent. Participants were periodontally examined and classified as follows: thirty patients with CP (15 females and 15 males, mean age: 42.6±9.1 years) and thirty individuals with clinically healthy periodontium served as controls (subjects without CP) (15 females and 15 males, mean age: 39.2±7.2).

A complete clinical examination was performed, as well as complete medical and dental histories. All participants of the group with and without CP were in good health and had not received periodontal therapy. Pregnant women, alcohol users, smokers, and individuals with any systemic chronic degenerative, autoimmune, infectious, or contagious disease or who were under pharmacological treatment were exclusion criteria for participants of both groups.

This study was approved by the Medical Ethics Review Committee of the University of Guadalajara. All participants were told the purpose of the study and accordingly signed the informed consent; they also answered a survey to determine if they met the inclusion criteria for the project.

### 2.2. Periodontal Diagnosis

Periodontal examination was executed by a single calibrate examiner Periodontist (ALML), who established the periodontal diagnosis, considering radiographic and clinical parameters. Periodontal examination includes full-mouth clinical measurements of the plaque index (PI), bleeding on probing (BOP), clinical attachment level (CAL), and probing pocket depth (PD). BOP that was the average of the percentage of sites evaluated with the presence of bleeding was recorded as the mean percentage of sites. CAL and PD were assessed at six sites per tooth. Diagnosis was based on the criteria proposed by the International Workshop for a Classification of Periodontal Diseases and Conditions [25].

### 2.3. Sample Preparation and Analysis

Samples of GCF, saliva, and serum were taken in the morning (from 10:00 to 12:00 am) and the participants were instructed not to eat and drink anything except water or use dental hygiene products at least two hours before their appointment.

Nonstimulated whole saliva samples were collected under resting conditions. Participants rinsed their mouths with water and, then, 3 mL of whole saliva was obtained by patients expectorating into disposable tubes [26]. Samples were refrigerated for transport, and then centrifuged at 10,000 g for 10 minutes at 4°C. The supernatant was separated (2.5 mL each) and a cocktail of protease inhibitor (Protease inhibitor Mix GE Healthcare Cat. No. 80650123) was added.

GCF samples were collected from a mesiobuccal and distobuccal site on each tooth (molars, premolars, canines, and incisors). In individuals with CP, GCF samples were obtained from sites with a depth of ≥5 CAL. In the group without CP, GCF samples were collected from teeth showing PD ≤3 without CAL or BOP. Three GCF samples were collected from each participant. The sites were gently dried and isolated with cotton and GCF was collected by the intracrevicular absorption method [27], with paper tips (PerioPaper Strep, Oraflow Inc, NY. USA). The paper tips were placed for one minute and the moisture density and volume of the GCF was calculated with an electronic micromoisture meter (Periotron 8000, Oraflow, Inc., NY equipment USA). Subsequently, the samples were eluted, and paper tips were placed and shaken for 30 minutes in a sterile phosphate buffer saline (PBS). Then, samples were centrifuged at 10,000 g for 10 minutes at 4°C in order to remove the dry paper tips.

The serum sample was obtained from whole blood by venipuncture and centrifuged at 1,972 g for 10 minutes at room temperature. The serum was separated and protease inhibitor was added. All the samples were stored at −80°C until analysis and a single freeze cycle was performed.

MIF quantification was made by sandwich ELISA method (BioLegend® Legend Max TM Human Active MIF, Cat. No. 438408) according to manufacturer's instructions. Absorbance was determined using a WHY101 microplate reader at a wavelength of 450 nm with a correction reading at 560 nm. MIF concentrations were calculated in ng/mL; these results were interpolated in a calibration curve of known concentrations included in the insert and the sensitivity of the kit was 8 pg/mL.

### 2.4. Statistical Analysis

Periodontal parameters are expressed as mean ± standard deviation (SD) and MIF concentrations as mean ± standard error (SE). All data were tested for normality using the Kolmogorov-Smirnov test. Differences in MIF concentrations were evaluated using Mann–Whitney's U test for intergroup comparisons and Wilcoxon test for intragroup comparisons. A Spearman correlation was performed to test the relationship between MIF concentration and periodontal clinical parameters. All tests were performed using the Statistical Program for Social Sciences (SPSS v11.0) for Windows (SPSS, Inc., Chicago, IL, USA). A p < 0.05 was considered statistically significant.

## 3. Result

### 3.1. Sociodemographic and Clinical Data

Sociodemographic and clinical periodontal parameters are summarized in Table 1. The age and gender of the participants between groups were not different or statistically significant. All clinical parameters (CAL, PD, BOP, and PI) were increased in individuals with CP compared with individuals without CP (p=0.001; Table 1).

### 3.2. MIF Concentrations in GCF, Saliva, and Serum

**Figure 1:**
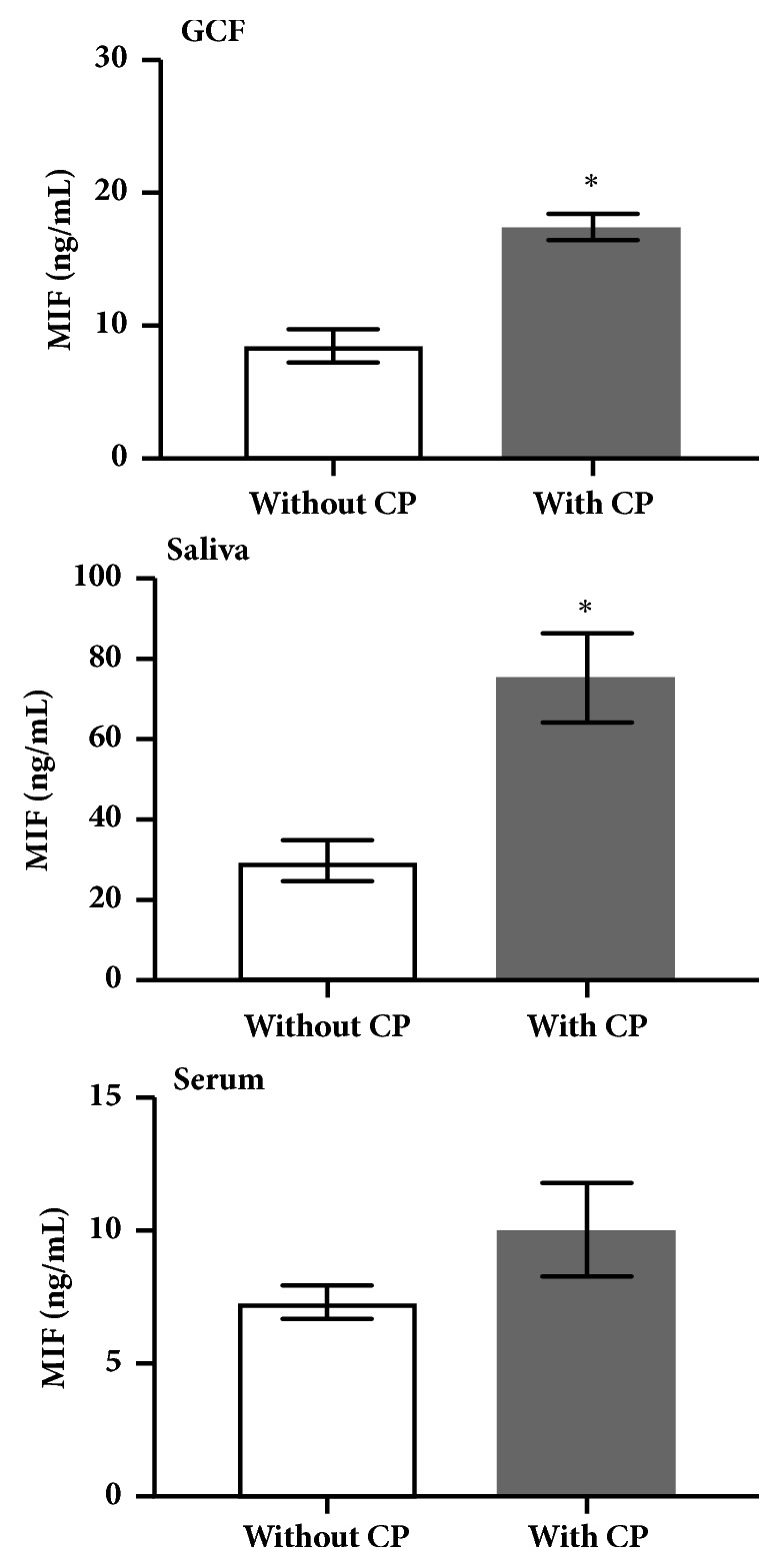
*Intergroup comparisons of MIF concentrations in GCF, saliva, and serum.* Data are expressed as mean ± standard error. CP: chronic periodontitis; GCF: gingival crevicular fluid; MIF: macrophage migration inhibitory factor; ng: nanogram; mL: milliliters. *∗*: statistically significant differences were considered with a p-value < 0.05.

MIF concentrations in subjects without CP in GCF, saliva, and serum were 8.85±1.28 ng/mL, 31.5±5.76 ng/mL, and 7.08±0.58 ng/mL, respectively, while in the group with CP, these values were 17.85±1.04 ng/mL, 72.94±10.90 ng/mL, and 9.91±1.65 ng/mL, respectively (Figure 1). MIF concentrations obtained in the three different fluids were compared between groups. Increased levels of MIF in GCF (p=0.001) and saliva (p=0.001) were found in the group with CP compared with the group without CP. Moreover, serum concentrations of MIF were higher in the group with CP, and no significant differences were observed between groups (p=0.509, Figure 1).

**Figure 2:**
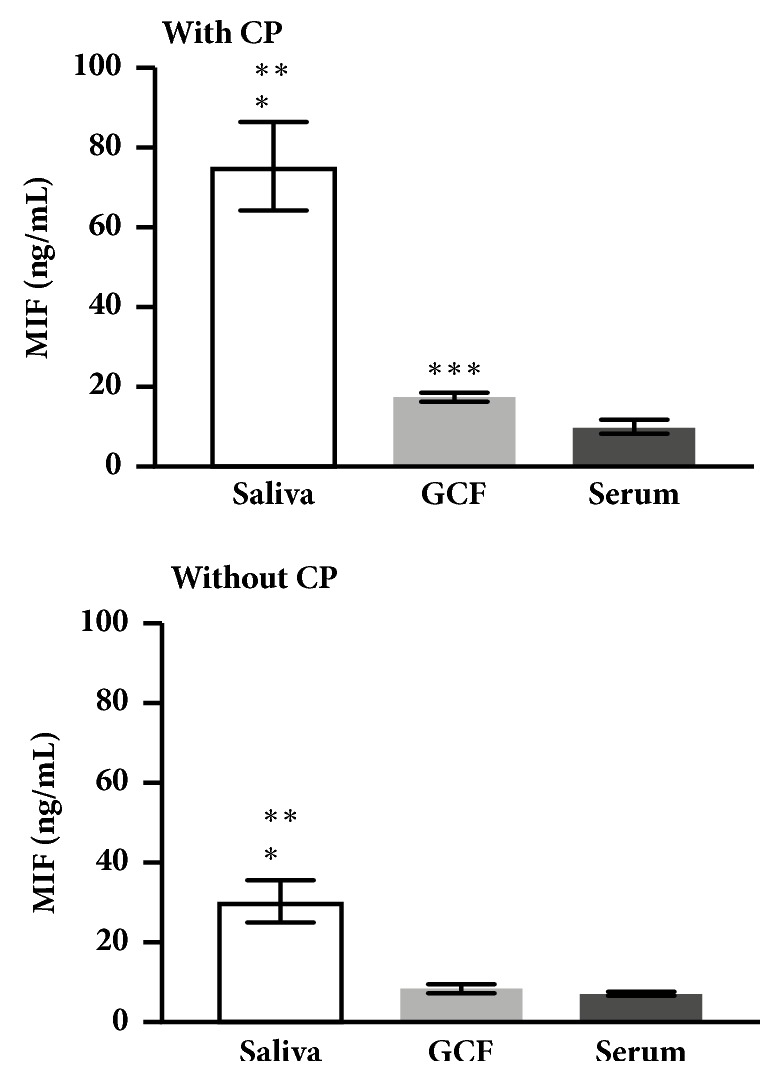
*Intragroup comparisons of MIF concentrations in GCF, saliva, and serum.* Data are expressed as mean ± standard error. CP: chronic periodontitis; GCF: gingival crevicular fluid; ng: nanogram; MIF: macrophage migration inhibitory factor; mL: milliliters. Intragroup comparisons were made *∗*saliva vs GCF, *∗∗* saliva vs serum and *∗∗∗*GCF vs serum; *∗*: p-value of <0.05 was considered statistically significant.

Intragroup comparisons of MIF concentrations were made between the three different fluids from each study group and we found that, in the group without CP, MIF levels were significantly higher in saliva (p=0.001) compared with GCF and serum (p=0.001) and no significant differences were observed between MIF levels in GCF and serum (Figure 2). However, in the group with CP, MIF levels in saliva were statistically higher compared with GCF (p=0.001) and serum (p=0.001) (Figure 2). Also, MIF levels in GCF were increased compared with serum (p=0.001, Figure 2).

### 3.3. Correlation

**Figure 3:**
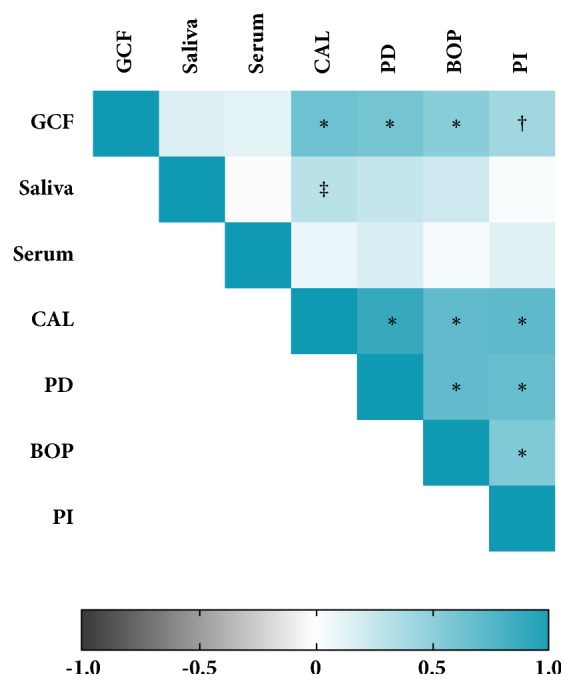
*Correlation heat map between GCF, salivary, and serum levels of MIF with clinical measurements.* CAL: clinical attachment level; PD: probing depth; PI: plaque index; BOP: bleeding of probing; MIF: macrophage migration inhibitor factor; GCF: gingival crevicular fluid. Spearman correlation; r: correlation coefficient. ^‡^p=0.034; ^†^p=0.01; ^*∗*^p= 0.001.

In order to associate MIF concentrations with clinical periodontal parameters, a positive significant correlation was found between MIF concentrations in GCF and clinical parameters (CAL: p=0.001; PD: p=0.001, BOP: p=0.001, and PI: p=0.004) and between MIF concentrations in saliva and CAL (p= 0.03) (Figure 3). No significant correlation was observed between MIF concentrations in serum and clinical parameters (Figure 3).

## 4. Discussion

MIF has been proposed as a key proinflammatory cytokine in the development of various inflammatory and autoimmune pathologies [17, 19, 28, 29].

In CP, the severity of the disease is associated with the unregulated production of proinflammatory cytokines [7].

Some studies have identified MIF overexpression in GCF from subjects with gingivitis [23], in gingival tissue from subjects with CP, in serum and tissue of mice with induced periodontitis [15, 30]. In addition, it has been described that MIF gene polymorphism (*MIF*-173 G/C) increased the risk of CP [31]. In our study we investigated MIF concentrations in GCF, saliva, and serum in individuals with CP and to correlate MIF concentrations with periodontal clinical parameters, with the aim of now MIF protein levels at the different biofluids.

We found higher MIF levels in GCF and saliva in individuals with CP. The high levels of MIF in GCF and saliva could be attributed to the characterized microbial change (from Gram-positive to Gram-negative bacterial strains) during the transition from health to periodontal disease [32, 33]. Therefore, characteristic bacterial endotoxins in the CP, such as LPS [6, 34], can stimulate the release of MIF in resident cells like epithelial cells, macrophages, or neutrophils [35, 36], present in tissues where the disease develops. The increase of MIF could also contribute with the unregulated production of proinflammatory cytokines that play a part in the severity of the disease [7]. On that account, it has been described in a periodontitis model in mice that MIF promote the activation of transcription factors that activate osteoclastogenesis [15].

In the present work, no significant correlation was observed between serum levels of MIF and CAL, PD, BOP, and PI. However, a positive correlation was found between GCF levels of MIF and all clinical measurements as well as between salivary levels of MIF and CAL. The overall correlation between GCF levels of MIF and all clinical parameters in this work suggests that MIF may play an important role in inflamed periodontal tissue since the oral cavity and periodontal tissue are exposed to a wide variety of oral bacteria [22, 37, 38].

GCF is obtained directly from the gingival sulcus where CP is developed. MIF concentration in GCF can be used as a biomarker of the progression of CP.

However, salivary levels of MIF did not correlate with clinical parameters except with CAL. In the present work, the concentration of this cytokine in saliva did not show a strong lineal relationship with clinical parameters; this could be explained since MIF concentration in saliva is heterogeneous combined with the periodontal disease and the fact that the concentration of MIF could be affected by environmental factors of the oral cavity, such as microbes.

MIF acts in an autocrine manner and promotes the release of other proinflammatory molecules (TNF, IFN-*γ*, IL-1*β*, IL-2, IL-6, IL-8, MIF, PGE2, and MMP) [10, 38]; these molecules increase inflammation further in the CP and thus the degradation of the periodontal tissue.

MIF has the ability to interact with chemokine receptors, such as CXCR2 [39], so that in CP, MIF also participate directly in the recruitment of leukocytes and monocytes and later promote their differentiation to mature osteoclasts as described in experimental studies [15].

What we can explain is that MIF is stimulus-dependent since dental plaque is the main etiological and persistent agent [40] because it maintains the activation of both the innate and the adaptive immune response and thus prolongs the release of MIF [9].

Serum levels of MIF were higher in the CP group, but no significant differences were found between the study groups. This could be explained since inflammatory mediators, bacteria, and endotoxins of CP can enter and travel through the circulation, because of the permeability of the gum due to the inflammation of periodontal tissue [41, 42].

The serum concentrations of MIF obtained in the present work in individuals without CP coincide with values reported as normal in clinically healthy subjects ranging from 2 to 6 ng/ml [43].

Furthermore, consider the intragroup comparisons to determine the behavior expressions of MIF in the biofluids: GCF (MIF local expressions), saliva (MIF semilocal expressions), and serum (MIF systemic expressions) of the study groups. We observed that the concentrations of MIF in saliva were significantly higher than in GCF and serum in both groups and that MIF GCF level was greater than serum in the group with CP. In the CP group it is justifiable that saliva and GCF present higher MIF levels than serum. This increase may be due to the main stimulus for the release of MIF is found in the oral cavity, which is the presence of the disease and the bacterial component that causes the inflammatory response. On the other hand, in individuals without CP the same tendency was observed as in the group with CP.

We hypothesized that this result may be due to the constitutive expression of MIF in cells that are in coating tissues, such as the mucosa, which is in direct contact with the external environment, so epithelial cells could be one of the main sources of MIF production [9]. In the oral cavity, the constitutive expression of MIF is crucial, since MIF can regulate the response of the host against to infections and stress [9].

In this study, our results show that the presence of CP per se increases the levels of MIF in GCF and saliva. These results can position MIF in the panel of cytokines that promotes the inflammatory response in CP. However, it is important to continue carrying out more studies to further elucidate the participation of this cytokine in the pathology of CP.

## 5. Conclusions

According to our results, it can be concluded that CP increases the concentrations of MIF in GCF and saliva. Furthermore, the association between MIF in GCF and clinical parameters suggests that MIF might be a biological marker of the severity of CP. This could reflect the participation of MIF in the microenvironment of the oral cavity.

## Figures and Tables

**Table 1:** Sociodemographic and periodontal parameters of the study groups.

	Groups		
Variables	(1) Without CP n=30	(2) With CP n=30	p value
*Gender*			
* (i) Female (*%)	15 (50)	15 (50)	
*(ii) Male (*%)	15 (50)	15 (50)	
*Age (years)*	39.2±7.2	42.6±9.1	ns
*CAL (mm)*	0.92±0.27	4.36±1.14	p= 0.001
*PD (mm)*	1.18±0.43	3.44±0.89	p= 0.001
*BOP (*%)	6.13±3.25	14.07±5.03	p= 0.001
*PI (*%)	12.37±5.62	33.43±15.86	p= 0.001

Data are expressed as mean (±SD). Gender is expressed as percentages. CP: chronic periodontitis; CAL: clinical attachment level; PD: Probing depth; PI: plaque index; BOP: bleeding of probing; MIF: Macrophage Migration Inhibitor Factor; GCF: gingival crevicular fluid; SD: standard deviation; ns: not significant. U de Mann Whitney test was used to compare periodontal parameters.

## Data Availability

The raw data required to reproduce these findings cannot be shared at this time as the data also forms part of an ongoing study.
